# Replacement dynamics and the pathogenesis of the Alpha, Delta and Omicron variants of SARS-CoV-2

**DOI:** 10.1017/S0950268822001935

**Published:** 2022-12-20

**Authors:** Thomas Ward, Alex Glaser, Christopher E. Overton, Bob Carpenter, Nick Gent, Anna C. Seale

**Affiliations:** 1 UK Health Security Agency, London, UK; 2 The Flatiron Institute, Center for Computational Mathematics, New York, NY, USA; 3 University of Warwick, Warwick Medical School - Health Sciences, Warwick, UK

**Keywords:** Epidemics, epidemiology, infectious disease, infectious disease epidemiology

## Abstract

New SARS-CoV-2 variants causing COVID-19 are a major risk to public health worldwide due to the potential for phenotypic change and increases in pathogenicity, transmissibility and/or vaccine escape. Recognising signatures of new variants in terms of replacing growth and severity are key to informing the public health response. To assess this, we aimed to investigate key time periods in the course of infection, hospitalisation and death, by variant. We linked datasets on contact tracing (Contact Tracing Advisory Service), testing (the Second-Generation Surveillance System) and hospitalisation (the Admitted Patient Care dataset) for the entire length of contact tracing in the England – from March 2020 to March 2022. We modelled, for England, time delay distributions using a Bayesian doubly interval censored modelling approach for the SARS-CoV-2 variants Alpha, Delta, Delta Plus (AY.4.2), Omicron BA.1 and Omicron BA.2. This was conducted for the incubation period, the time from infection to hospitalisation and hospitalisation to death. We further modelled the growth of novel variant replacement using a generalised additive model with a negative binomial error structure and the relationship between incubation period length and the risk of a fatality using a Bernoulli generalised linear model with a logit link. The mean incubation periods for each variant were: Alpha 4.19 (95% credible interval (CrI) 4.13–4.26) days; Delta 3.87 (95% CrI 3.82–3.93) days; Delta Plus 3.92 (95% CrI 3.87–3.98) days; Omicron BA.1 3.67 (95% CrI 3.61–3.72) days and Omicron BA.2 3.48 (95% CrI 3.43–3.53) days. The mean time from infection to hospitalisation was for Alpha 11.31 (95% CrI 11.20–11.41) days, Delta 10.36 (95% CrI 10.26–10.45) days and Omicron BA.1 11.54 (95% CrI 11.38–11.70) days. The mean time from hospitalisation to death was, for Alpha 14.31 (95% CrI 14.00–14.62) days; Delta 12.81 (95% CrI 12.62–13.00) days and Omicron BA.2 16.02 (95% CrI 15.46–16.60) days. The 95th percentile of the incubation periods were: Alpha 11.19 (95% CrI 10.92–11.48) days; Delta 9.97 (95% CrI 9.73–10.21) days; Delta Plus 9.99 (95% CrI 9.78–10.24) days; Omicron BA.1 9.45 (95% CrI 9.23–9.67) days and Omicron BA.2 8.83 (95% CrI 8.62–9.05) days. Shorter incubation periods were associated with greater fatality risk when adjusted for age, sex, variant, vaccination status, vaccination manufacturer and time since last dose with an odds ratio of 0.83 (95% confidence interval 0.82–0.83) (*P* value < 0.05). Variants of SARS-CoV-2 that have replaced previously dominant variants have had shorter incubation periods. Conversely co-existing variants have had very similar and non-distinct incubation period distributions. Shorter incubation periods reflect generation time advantage, with a reduction in the time to the peak infectious period, and may be a significant factor in novel variant replacing growth. Shorter times for admission to hospital and death were associated with variant severity – the most severe variant, Delta, led to significantly earlier hospitalisation, and death. These measures are likely important for future risk assessment of new variants, and their potential impact on population health.

## Introduction

New SARS-CoV-2 variants causing COVID-19 are a major concern to public health worldwide, due to the potential for increases in incidence and infection severity. Understanding the characteristics of these new variants is critical, to inform the assessment of risk. In the UK, epidemic waves of novel variants have been associated with replacement: wild-type was replaced by Alpha (B.1.1.7) [1] at the end of 2020; Delta (B.1.617.2) replaced Alpha by July 2021 [2]; Omicron BA.1 (B.1.1.529) replaced Delta in December 2021 [3] and Omicron BA.2 (B.1.1.529.2) became the dominant variant in March 2022 [4].

Alpha was first detected in the UK in November 2020 from a sample collected in England on 20th September 2020 [5] and became the dominant variant in the second wave of the SARS-CoV-2 epidemic. Alpha has three residue mutations on the spike protein [6], impacting the conformity of the receptor binding domain (RBD), which may have increased infectivity [7] relative to wild-type. Alpha could be detected in the UK through deletions at 69–70 positions, which result in S-gene target failure in diagnostic clinical reverse transcription-polymerase chain reaction (RT-PCR) tests. Alpha was found to be more severe than wild-type, with a 73% higher hazard of mortality [8].

Delta was identified in India in October 2020 [9], a sub-lineage of B.1.617, and became the dominant variant in India from early 2021 [10]. In March 2021, the first detected case of Delta was identified in England, followed by sustained exponential growth, and the subsequent decay in Alpha. There were seven important mutations on the spike protein of Delta relative to the earlier D614G variant; two in the RBD and in the N terminal domain, then a further five that may contribute towards immunological evasion [11]. In surveillance population studies, evidence of increased hospitalisation risk for unvaccinated individuals infected with Delta relative to Alpha was found [12]. Moreover, a reduction in vaccine effectiveness for Delta was observed when contrasted with Alpha [13]. A sub-lineage of Delta (designated as AY.4.2 or Delta Plus) increased in England subsequently, and by the 12th November accounted for 11.2% of all Delta cases that were sequenced [14]. This variant had two spike mutations (A222V and Y145H) which was thought may lead to a transmission advantage over Delta.

The B.1.1.529 variant, named Omicron, was reported to the World Health Organization on the 24th November 2021 [15]. It was initially detected from specimens collected in South Africa [16] on the 14th November 2021. Omicron had the greatest number of mutations for any sequenced variant relative to the wild-type strain [17]. Studies have found over 50 mutations and at least 32 in the spike protein [18, 19], which is roughly double the number seen in Delta. Different subvariants of Omicron have caused epidemic growth in the UK, including BA.1 and BA.2, but more recently BA.4 and BA.5 [20]. Another recent study found 39 mutations in BA.1 and 31 in BA.2 of which 21 are shared [21]. A reduction in the severity of Omicron infections has been observed with a reduced hospitalisation risk [22] relative to earlier dominant variants, which may be related to increased replication of Omicron in the bronchi [23] and less in the lower lung parenchyma. However, there are conflicting reports regarding the severity for BA.2 relative to BA.1 [24, 25] where population level analysis will be hindered by the high attack rate of a preceding antigenically similar variant.

Recognising signatures of new variants in terms of replacing growth and severity are key to informing the public health response. To assess potential signatures and variant characteristics we invesitigated key time periods in the course of infection, hospitalisation and death.

## Methods

We assessed the variation between the Alpha, Delta, Delta Plus, Omicron BA.1 and Omicron BA.2 variants in terms of clinical time delay distributions through Bayesian doubly interval censored modelling. We modelled growth rates through generalised additive modelling (GAM), with a negative binomial error structure, and variant proportions with binomial uncertainty to assess replacement rates. The relationship between incubation period length and the risk of a fatality was modelled using a Bernoulli generalised linear model (GLM) with a logit link. Through the algorithmic linkage of contact tracing and clinical data, we calculated the incubation period, the time from hospitalisation to death and infection to hospitalisation for each variant. This process is described in more detail below.

### Epidemiological and clinical data

The Contact Tracing Advisory Service (CTAS) dataset is collected by the Department of Health and Social Care and records data on confirmed SARS-CoV-2 cases, and the information required for contact tracing. For this study we extracted the contact imputed exposure date, which is the most likely day of exposure estimated by the contact tracer or individual reporting the event. The cases and contacts are linked by a matching identifier to create transmission branch pairs.

The National Pathology Exchange (NPEx) dataset includes data from laboratories on pathology results and tests requested in the UK. NPEx includes information on the symptom onset of confirmed COVID-19 cases which we extracted. The Second-Generation Surveillance System (SGSS) dataset includes information on laboratory notifications and isolates. From SGSS we extracted the results of whole-genome sequencing and genotyping for SARS CoV-2 RT-PCR tests, which is available for a subset of positive tests. The CTAS, SGSS and NPEx datasets were then algorithmically linked by a pseudo identifier in SQL for the contact imputed exposure and symptom onset dates by SARS-CoV-2 variant.

The Admitted Patient Care (APC) dataset includes all activity for NHS Trusts, NHS Foundation Trusts and institutions where care is NHS funded, and is derived from the NHS Secondary Uses Service. Within this dataset an admission to one hospital is defined as a ‘spell’, which is further divided by ‘episodes’ that are defined as a period of care that a patient receives [26]. For this study the first episode for each spell was used for individuals that had multiple episodes. We further extracted for hospitalised individuals the date of death from this spell. The CTAS, APC, SGSS and NPEx datasets were algorithmically linked by a pseudo-identifying number to acquire the date of exposure, date of symptom onset, date of hospitalisation and date of death by SARS-CoV-2 variant.

### Time delay distribution modelling

The analysis was conducted on the entire length of the contact tracing data which was operational from 28th May 2020 to 23rd February 2022. From the linked datasets the primary dates of interest for this analysis were exposure, symptom onset, hospitalisation and mortality, which were employed to measure the incubation period, the time from infection to hospitalisation and the time from hospitalisation to death. The incubation period was defined as the time from contact imputed exposure date to symptom onset date and this study therefore excludes asymptomatic cases. Analysis of the time from hospitalisation to death and infection to hospitalisation was not conducted for Delta Plus and Omicron BA.2 variant due to an inadequate sample size available.

The modelling methodology draws on Ward and Johnsen [27] and Reich *et al*. [28] utilising a doubly interval censored modelling approach for coarsely recorded data. Two events *A* and *B* occur at times *α* and *β* that are not precisely known, *α* ∈ [*α*_−_, *α*_+_], *β* ∈ [*β*_−_, *β*_+_]. Let the time between the two events be *T*, a continuous random variable with probability density function *f*(*t*;*θ*), dependent on parameters *θ*. The joint probability of both events is
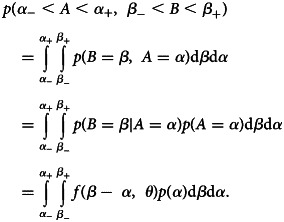


In the absence of information informing *p*(*α*) over short time windows, let it be a uniform distribution between *α*_−_ and *α*_+_.

The likelihood of *θ* and an observed data point *X*^*i*^ can then be defined, as
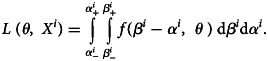


Then for multiple data points *X* = {*X*^*i*^}, the defined likelihood is
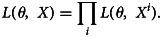


Within the context of this paper, the events *α* and *β* refer to:

**Table:** 

Events		
Time Period	*α*	*β*
Incubation period	Exposure	Symptom onset
Infection to hospitalisation	Exposure	Hospital admission
Hospitalisation to death	Hospital admission	Death

The probability density function *f* is taken to follow the Gamma, lognormal and Weibull distributions. To compare the model fits we calculate the leave-one-out cross validation [29] through Pareto-smoothed importance sampling. We then compared the model fits through the expected log pointwise predictive density. To fit the model to the data, through the Cmdstanr package [30], we used Markov chain Monte Carlo (MCMC) and evaluated its convergence using potential scale reduction factor [31] or 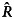 where it is desirable to have a value less than 1.01. The models used weakly informative priors that were chosen to penalise unrealistic parameters. The MCMC output provided the posterior distribution of the parameters. We then calculated from this posterior distribution the credible intervals (CrIs) that are reported for the mean, standard deviation (s.d.) and the percentiles of the distribution.

To limit the inclusion of non-COVID-related mortality the time from hospitalisation to death was restricted to 50 days [32]. To reduce the likelihood of incorrect contact tracing pairing events we include values up to and including 15 days from exposure to symptom onset and 30 days from exposure date to hospitalisation date. These definitions were over the 95th percentile of the time delay distributions prior to the application of the exclusion criteria.

### Bernoulli GLM with a logit link

To estimate the impact of the length of the incubation period on the likelihood of a mortality we developed a Bernoulli GLM with a logit link. Variable interactions were explored through directed acyclic graphs prior to the sensitivity analysis of the covariates. The final model was selected based on the lowest Akaike information criterion value and Bayesian information criterion:
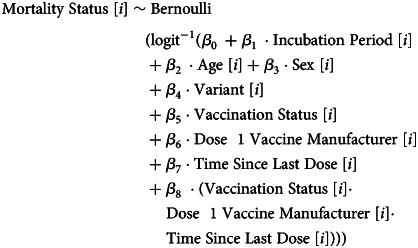


We calculated the odds ratio and probability of a fatality occurring given a step change in the incubation period.

### GAM with a negative binomial error structure and binomial probability analysis

To estimate the time varying growth rates for each variant we used a method described by Ward *et al*. [2]. Estimating the exponential growth rate requires an assumption of an exponential structure to the data. In a phase of constant exponential growth, we can approximate an epidemic by using *y*(*t*) = *y*(0)*e*^*rt*^, where *y*(0) is the initial number of cases and *r* is the exponential growth rate. This can be generalised to an epidemic which is not in the exponential phase through replacing *rt* with a smooth function of time *s*(*t*). This smooth function is then estimated through a generalised additive model with a canonical log-link and negative binomial error structure. The model was fit to the specimen date of the RT-PCR positive whole-genome sequenced and genotyped cases. We used cubic regression splines, the optimal number of knots was calculated through sensitivity analysis, and random effects were included on day of week to account for the cyclical nature of reported positive tests. Then for this model (*y*(*t*) = *y*(0)*e*^*s*(*t*)^), the number of cases at time *t*, *y*(*t*), is proportional to the exponential of the smooth function with time, exp(*s*(*t*)). The time derivative of the smoother d*s*(*t*)/d*t* is thus the instantaneous growth rate, *r*_*s*_, and the doubling times can be understood as *t*_*D*_ = log (2)/*r*_*s*_. To assess the rates of replacement, analysis was conducted for the time varying proportions of whole-genome sequenced and genotyped RT-PCR tests for each variant and we used the method from Wilson [33] to calculate binomial uncertainty.

### Statistical tests

To contrast the posterior distributions of each variant we calculated pairwise *t* tests using a Bonferroni correction [34]. Finally, to assess the impact of the varying age distributions between each sample chi-squared tests were conducted.

## Results

### Incubation period

**Fig. 1.**
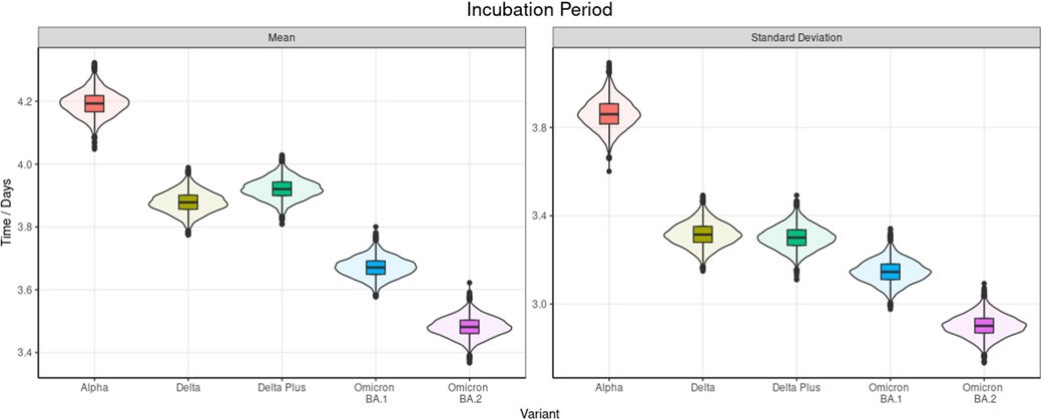
Violin and box and whisker plot of the posterior estimates of the mean and s.d. of the doubly interval censored modelled fit to a lognormal distribution for the incubation period. This includes data from September 2020 to March 2022.

The shortest incubation period to date was found for Omicron BA.2, at 3.48 (95% CrI 3.43–3.53) days. Each successive replacing variant had a shorter incubation period relative to the previously dominant variant, with *P* values indicating statistically distinct posterior distributions (*H*_1_) (full analysis is provided in Supplementary A). Delta had a shorter incubation period by 0.32 (95% CrI 0.2–0.44) days relative to Alpha, with a relative percentage reduction of 7.64% (95% CrI 4.84–10.33) in the posterior mean. Omicron BA.1 had a shorter incubation period by 0.2 (95% CrI 0.10–0.32) days relative to Delta and a difference of 5.17% (95% CrI 2.62–8.14) in the posterior mean. The Omicron BA.2 variant had a shorter incubation period by 0.19 (95% CrI 0.08–0.29) days relative to Omicron BA.1 with a relative reduction of 5.18% (95% CrI 2.21–7.80) in the posterior mean. We further found there was a reduced s.d. for replacing variants (Fig. 1). The Delta and Delta Plus variants had almost entirely overlapping CrIs (*P* value = 1) for the posterior distributions of the mean.

Incubation period data had the best fit using a lognormal distribution in the modelling. The results across all ages can be seen in Table 1. The age distribution of the samples can be seen in Supplementary B and the chi-squared test results did not indicate strong evidence of a difference (*P* value 0.90).

**Table 1.** Posterior estimates of the mean and s.d. of the doubly interval censored modelled fit to a lognormal distribution for all ages by variant, 95% CrIs are provided and the 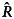 of the mean

Incubation period for all ages							
Variant	*N*	Distribution	Mean	s.d.	*α*	*β*	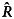 (mean)
Alpha	47 808	Lognormal	4.19 (4.13–4.26)	3.86 (3.75–3.97)	1.13 (1.11–1.14)	0.78 (0.77–0.79)	1.00
Delta	271 628	Lognormal	3.87 (3.82–3.93)	3.32 (3.23–3.40)	1.08 (1.07–1.09)	0.74 (0.73–0.75)	1.00
Delta Plus	19 594	Lognormal	3.92 (3.87–3.98)	3.30 (3.22–3.39)	1.10 (1.09–1.11)	0.73 (0.72–0.74)	1.00
Omicron BA.1	116 163	Lognormal	3.67 (3.61–3.72)	3.14 (3.06–3.22)	1.03 (1.01–1.04)	0.74 (0.73–0.75)	1.00
Omicron BA.2	8785	Lognormal	3.48 (3.43–3.53)	2.90 (2.82–2.98)	0.98 (0.97–1.00)	0.73 (0.72–0.73)	1.00

For each variant, we find substantial overlap between the CrIs across the age groups (Tables 2–6), which is indicative of non-significant variation by age. For each age group, we find that replacing variants have a shorter incubation period, which is consistent with the all-ages analyses.

**Table 2.** Posterior estimates of the mean and s.d. of the doubly interval censored modelled fit to a lognormal distribution for Alpha by age groups, 95% CrIs are provided and the 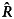 of the mean

Alpha incubation period by age group							
Age group	*N*	Distribution	Mean	s.d.	*α*	*β*	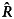 (mean)
0–19	9569	Lognormal	4.14 (4.08–4.21)	3.74 (3.64–3.85)	1.12 (1.11–1.14)	0.77 (0.76–0.78)	1.00
20–39	16 616	Lognormal	4.17 (4.10–4.24)	3.84 (3.73–3.96)	1.12 (1.10–1.14)	0.78 (0.77–0.79)	1.00
40–59	15 323	Lognormal	4.18 (4.11–4.26)	3.81 (3.69–3.94)	1.13 (1.09–1.12)	0.78 (0.77–0.79)	1.00
≥60	6300	Lognormal	4.27 (4.17–4.38)	3.61 (3.64–3.99)	1.16 (1.14–1.18)	0.77 (0.75–0.78)	0.99

**Table 3.** Posterior estimates of the mean and s.d. of the doubly interval censored modelled fit to a lognormal distribution for Delta Plus by age groups, 95% CrIs are provided and the 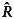 of the mean

Delta Plus incubation period by age group							
Age group	*N*	Distribution	Mean	s.d.	*α*	*β*	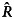 (mean)
0–19	3863	Lognormal	3.93 (3.88–3.99)	3.23 (3.15–3.32)	1.11 (1.10–1.12)	0.72 (0.71–0.73)	1.00
20–39	4443	Lognormal	3.84 (3.77–3.92)	3.24 (3.12–3.37)	1.08 (1.06–1.10)	0.73 (0.72–0.75)	1.00
40–59	7764	Lognormal	3.85 (3.79–3.91)	3.13 (3.05–3.22)	1.09 (1.08–1.11)	0.71 (0.70–0.72)	1.00
≥60	3524	Lognormal	3.83 (3.74–3.92)	3.22 (3.08–3.36)	1.08 (1.05–1.10)	0.73 (0.71–0.75)	1.00

**Table 4.** Posterior estimates of the mean and s.d. of the doubly interval censored modelled fit to a lognormal distribution for Delta by age groups, 95% CrIs are provided and the 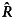 of the mean

Delta incubation period by age group							
Age group	*N*	Distribution	Mean	s.d.	*α*	*β*	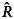 (mean)
0–19	55 032	Lognormal	3.86 (3.81–3.92)	3.39 (3.30–3.48)	1.07 (1.05–1.08)	0.76 (0.75–0.77)	1.00
20–39	73 453	Lognormal	3.84 (3.79–3.90)	3.26 (3.18–3.35)	1.07 (1.06–1.09)	0.74 (0.73–0.75)	1.00
40–59	97 083	Lognormal	3.90 (3.85–3.95)	3.17 (3.09–3.25)	1.11 (1.09–1.12)	0.71 (0.70–0.72)	1.00
≥60	46 060	Lognormal	3.83 (3.78–3.88)	3.25 (3.17–3.34)	1.07 (1.06–1.08)	0.74 (0.73–0.75)	1.00

**Table 5.** Posterior estimates of the mean and s.d. of the doubly interval censored modelled fit to a lognormal distribution for Omicron BA.1 by age groups, 95% CrIs are provided and the 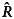 of the mean

Omicron BA.1 incubation period by age group							
Age group	*N*	Distribution	Mean	s.d.	*α*	*β*	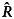 (mean)
0–19	17 319	Lognormal	3.77 (3.71–3.82)	3.36 (3.27–3.45)	1.03 (1.02–1.05)	0.77 (0.76–0.78)	1.00
20–39	39 400	Lognormal	3.64 (3.58–3.69)	3.20 (3.12–3.29)	1.00 (0.99–1.02)	0.76 (0.75–0.77)	1.00
40–59	39 618	Lognormal	3.70 (3.65–3.75)	3.24 (3.15–3.33)	1.02 (1.01–1.04)	0.76 (0.74–0.77)	1.00
≥60	19 826	Lognormal	3.76 (3.71–3.82)	3.25 (3.16–3.33)	1.05 (1.03–1.06)	0.75 (0.74–0.76)	1.00

**Table 6.** Posterior estimates of the mean and s.d. of the doubly interval censored modelled fit to a lognormal distribution for Omicron BA.2 by age groups, 95% CrIs are provided and the 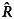 of the mean

Omicron BA.2 incubation period by age group							
Age group	*N*	Distribution	Mean	s.d.	*α*	*β*	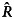 (mean)
0–19	1550	Lognormal	3.59 (3.45–3.73)	3.35 (3.12–3.60)	0.96 (0.93–1.00)	0.79 (0.77–0.82)	1.00
20–39	2710	Lognormal	3.41 (3.32–3.49)	2.71 (2.58–2.85)	0.98 (0.96–1.00)	0.70 (0.68–0.72)	1.00
40–59	3115	Lognormal	3.50 (3.42–3.58)	2.88 (2.75–3.02)	0.99 (0.97–1.02)	0.72 (0.70–0.74)	1.00
≥60	1410	Lognormal	3.46 (3.34–3.58)	2.80 (2.62–3.00)	0.99 (0.96–1.02)	0.71 (0.69–0.74)	1.00

Unvaccinated individuals had a shorter incubation period for each variant (Table 7), although they overlapped with the CrIs for vaccinated individuals. For every level of vaccination status, we find the incubation periods of replacing variants to be shorter than the preceding dominant variant, similar to the other analyses.

**Table 7.** Posterior estimates of the mean and s.d. of the doubly interval censored modelled fit to a lognormal distribution for each variant by vaccination status, 95% CrIs are provided and the 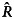 of the mean

Incubation period by vaccination status								
Vaccination status	Variant	*N*	Distribution	Mean	s.d.	*α*	*β*	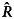 (mean)
Unvaccinated	Alpha	17 583	Lognormal	4.10 (4.04–4.17)	3.78 (3.67–3.89)	1.10 (1.09–1.12)	0.79 (0.77–0.80)	1.00
Unvaccinated	Delta	103 667	Lognormal	3.83 (3.77–3.88)	3.40 (3.31–3.49)	1.05 (1.04–1.06)	0.76 (0.75–0.77)	1.00
Unvaccinated	Delta Plus	7610	Lognormal	3.82 (3.76–3.89)	3.36 (3.25–3.47)	1.05 (1.04–1.07)	0.76 (0.75–0.77)	1.00
Unvaccinated	Omicron BA.1	38 106	Lognormal	3.59 (3.53–3.64)	3.21 (3.13–3.30)	0.98 (0.97–1.00)	0.77 (0.76–0.78)	1.00
Unvaccinated	Omicron BA.2	2221	Lognormal	3.28 (3.18–3.38)	2.88 (2.73–3.06)	0.9 (0.87–0.93)	0.76 (0.74–0.78)	1.00
One dose	Alpha	2248	Lognormal	4.21 (4.02–4.40)	3.91 (3.59–4.27)	1.13 (1.08–1.17)	0.79 (0.76–0.82)	1.00
One dose	Delta	18 068	Lognormal	3.90 (3.85–3.96)	3.39 (3.30–3.48)	1.08 (1.07–1.09)	0.75 (0.74–0.76)	1.00
One dose	Delta Plus	1327	Lognormal	3.98 (3.82–4.15)	3.48 (3.23–3.76)	1.10 (1.06–1.13)	0.75 (0.73–0.78)	1.00
One dose	Omicron BA.1	6866	Lognormal	3.75 (3.69–3.82)	3.25 (3.14–3.36)	1.04 (1.03–1.06)	0.75 (0.74–0.76)	1.00
One dose	Omicron BA.2	297	Lognormal	3.59 (3.35–3.86)	2.70 (2.35–3.10)	1.05 (0.99–1.12)	0.67 (0.62–0.72)	1.00
Two doses	Alpha	8330	Lognormal	4.11 (4.02–4.21)	3.77 (3.62–3.93)	1.11 (1.09–1.13)	0.78 (0.77 0.80)	1.00
Two doses	Delta	39 162	Lognormal	3.86 (3.81–3.91)	3.22 (3.14–3.30)	1.09 (1.07–1.10)	0.73 (0.72–0.74)	1.00
Two doses	Delta Plus	2692	Lognormal	3.96 (3.86–4.08)	3.27 (3.10–3.45)	1.12 (1.09–1.14)	0.72 (0.70–0.74)	1.00
Two doses	Omicron BA.1	19 279	Lognormal	3.66 (3.61–3.71)	3.15 (3.07–3.24)	1.02 (1.01–1.03)	0.75 (0.74–0.75)	1.00
Two doses	Omicron BA.2	1266	Lognormal	3.33 (3.22–3.44)	2.38 (2.23–2.55)	0.99 (0.96–1.03)	0.64 (0.62–0.67)	1.00
Three doses	Alpha	19 647	Lognormal	4.19 (4.12–4.25)	3.82 (3.72–3.93)	1.13 (1.12–1.14)	0.78 (0.77–0.79)	1.00
Three doses	Delta	110 731	Lognormal	3.89 (3.84–3.94)	3.11 (3.03–3.19)	1.11 (1.10–1.12)	0.70 (0.69–0.71)	1.00
Three doses	Delta Plus	7965	Lognormal	3.89 (3.83–3.95)	3.13 (3.04–3.22)	1.11 (1.09–1.12)	0.71 (0.70–0.72)	1.00
Three doses	Omicron BA.1	51 912	Lognormal	3.71 (3.66–3.76)	3.14 (3.06–3.22)	1.04 (1.03–1.06)	0.73 (0.72–0.74)	1.00
Three doses	Omicron BA.2	5001	Lognormal	3.61 (3.54–3.68)	3.05 (2.94–3.16)	1.01 (1.00–1.03)	0.73 (0.72–0.75)	1.00

### Incubation period and severity

**Fig. 2.**
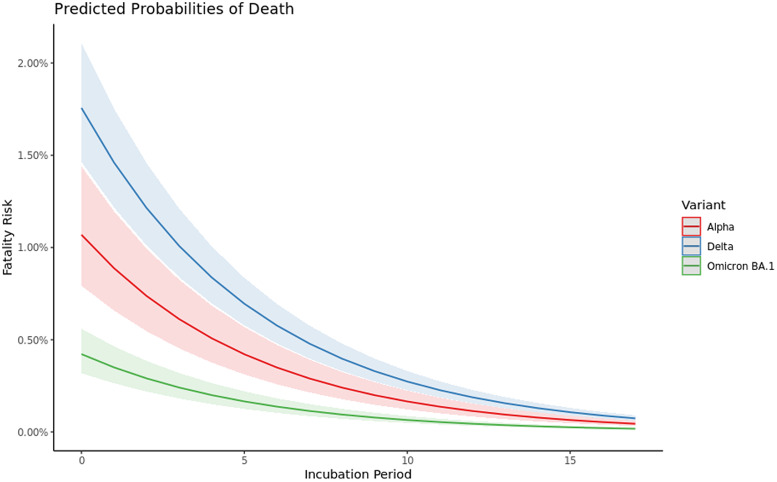
The marginal effects for the incubation period of the unvaccinated baseline factor, modelled using a Bernoulli GLM with a logit link. This includes data from October 2020 to March 2022. A sample size of 4 367 862 individuals.

We included a sample of 4 367 862 individuals to assess the incubation period length by outcome. Through Bernoulli GLM modelling we found a shorter incubation period was associated with an increased risk of death (*P* < 0.05), after adjusting for age, sex, variant, vaccination status, vaccination manufacturer and time since last dose (Fig. 2) with an odds ratio of 0.83 (95% confidence interval (CI) 0.82–0.83). The fatality risks for unvaccinated individuals who became symptomatic on the day of exposure were for Delta 1.76% (95% CI 1.46–2.11), Alpha 1.07% (95% CI 0.79–1.44) and Omicron BA.1 0.42% (95% CI 0.32–0.56). From the 10th day, after exposure, the risk begins to converge, in absolute terms, to 0.32% (95% CI 0.27–0.40), 0.19% (95% CI 0.15–0.27) and 0.08% (95% CI 0.06–0.10) for Delta, Alpha and Omicron BA.1, respectively.

### Cumulative distribution of the incubation period

**Fig. 3.**
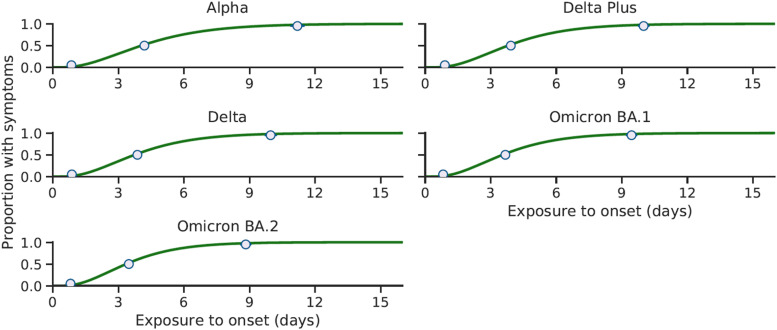
The posterior cumulative distribution for the incubation periods of the Alpha, Delta, Delta Plus, Omicron BA.1 and Omicron BA.2 variants. The median, 5th and 95th percentiles have been plotted. This includes data from September 2020 to 23rd February 2022.

The cumulative distribution function of the incubation period for the Alpha, Delta, Delta Plus, Omicron BA.1 and Omicron BA.2 variants can be seen in Fig. 3, which includes the median, 5th and 95th percentiles of the distribution. The 95th percentile of the incubation periods for each variant: Alpha, 11.19 (95% CrI 10.92–11.48) days; Delta, 9.97 (95% CrI 9.73–10.21) days; Delta Plus, 9.99 (95% CrI 9.78–10.24) days; Omicron BA.1, 9.45 (95% CrI 9.23–9.67) days and Omicron BA.2, 8.83 (95% CrI 8.62–9.05) days.

### Infection to hospitalisation

**Fig. 4.**
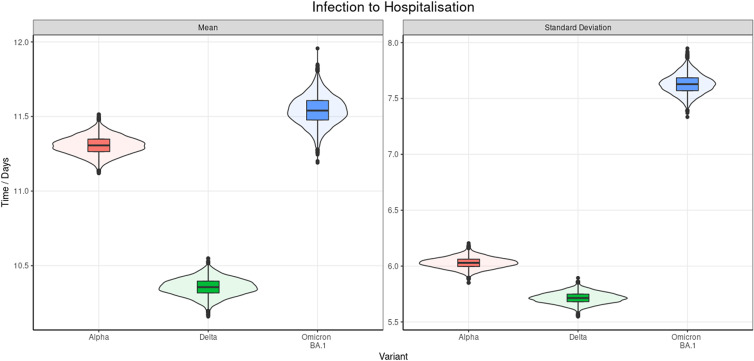
Violin and box and whisker plot of the posterior estimates for the mean and s.d. of the doubly interval censored modelled fit to a Weibull distribution for the time from infection to hospitalisation. This includes data from September 2020 to March 2022.

We found the time from infection to hospitalisation fits best to a Weibull distribution (Fig. 4 and Table 8). Delta had the shortest time interval from exposure to hospital admission with a mean of 10.36 (95% CrI 10.26–10.45) days, which was 0.95 (95% CrI 0.75–1.15) days and 1.18 (95% CrI 0.93–1.44) days shorter than Alpha and Omicron BA.1, respectively. Omicron BA.1 had the longest observed time from infection to hospitalisation and the mean of the posterior distribution was 0.23 (95% CrI 0.18–0.29) days longer than Alpha. The full pairwise tests with Bonferroni correction can be seen in Supplementary A and the results are strong evidence that the posterior distributions are distinct. The samples' age distributions for each variant can be seen in Supplementary C and the results of the chi-squared test found a non-significant *P* value of 0.85.

**Table 8.** Posterior estimates of the mean and s.d. of the doubly interval censored modelled fit to a Weibull distribution for the time from infection to hospitalisation for all ages by variant, 95% CrIs are provided and 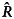 the of the mean

Infection to hospitalisation for all ages							
Variant	*N*	Distribution	Mean	s.d.	*α*	*β*	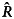 (mean)
Alpha	13 711	Weibull	11.31 (11.20–11.41)	6.03 (5.95–6.11)	1.96 (1.93–1.98)	12.75 (12.64–12.87)	1.00
Delta	15 650	Weibull	10.36 (10.26–10.45)	5.72 (5.64–5.80)	1.88 (1.86–1.91)	11.67 (11.56–11.78)	1.00
Omicron BA.1	6137	Weibull	11.54 (11.38–11.70)	7.623 (7.49–7.77)	1.54 (1.52–1.57)	12.83 (12.64–13.01)	0.99

### Hospitalisation to death

**Fig. 5.**
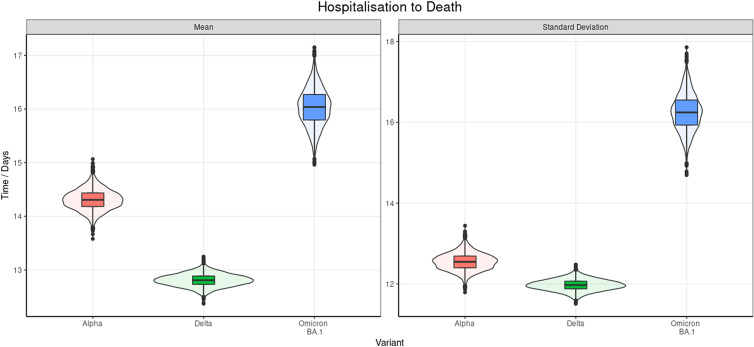
Violin and box and whisker plot of the posterior estimates of the mean and s.d. of the doubly interval censored modelled fit to a Weibull distribution for the time from hospitalisation to death. This includes data from September 2020 to March 2022.

We found the time from hospitalisation to death also best fits a Weibull distribution (Fig. 5 and Table 9). Similarly, to the time from exposure to hospital admission, Delta also had the shortest time from hospital admission to the reported date of death. We found the mean was 1.5 (95% CrI 1.00–2.00) days and 3.21 (95% CrI 2.46–3.98) days longer for Alpha and Omicron BA.1, respectively. We also further found a substantial divergence between Omicron BA.1 and Alpha of 1.71 (95% CrI 0.84–2.60) days. The results in Supplementary A illustrate the distinction between the posterior distributions of each variant was highly significant. The age distribution for the samples can be seen in Supplementary D and the chi-squared test found a non-significant *P* value of 0.88.

**Table 9.** Posterior estimates of the mean and s.d. of the doubly interval censored modelled fit to a Weibull distribution for the time from the time from hospitalisation to death across all ages by variant, 95% CrIs are provided and the 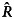 of the mean

Hospitalisation to death for all ages							
Variant	*N*	Distribution	Mean	s.d.	*α*	*β*	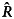 (mean)
Alpha	4353	Weibull	14.31 (14.00–14.62)	12.55 (12.22–12.90)	1.14 (1.21–1.66)	15.01 (14.66–15.36)	1.00
Delta	10 757	Weibull	12.81 (12.62–13.00)	11.97 (11.74–12.21)	1.07 (1.06–1.00)	13.15 (12.95–13.36)	1.00
Omicron BA.1	2107	Weibull	16.02 (15.46–16.60)	16.25 (15.51–17.02)	0.99 (0.96–1.02)	15.94 (15.34–16.53)	1.00

### Variant replacement and doubling times

**Fig. 6.**
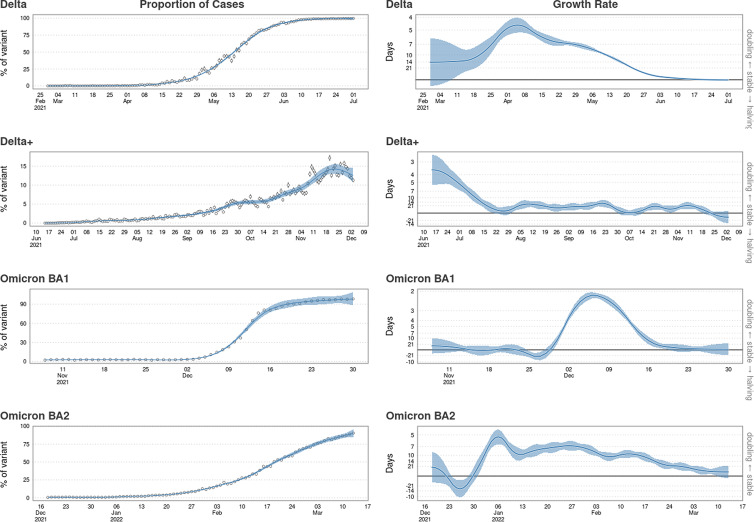
The temporal proportions with binomial CIs and the doubling times of the Delta, Delta Plus, Omicron BA.1 and Omicron BA.2 variants modelled using a GAM with a negative binomial error structure.

Doubling times and the proportion of variant cases recorded, until saturation, are shown in Fig. 6. From the first recorded variant case of Delta, replacement (of Alpha) took 124 days; the replacement of Delta by Omicron BA.1 took 53 days and the replacement of Omicron BA.1 by Omicron BA.2 took 86 days. Omicron BA.1 had the shortest replacement period and a peak doubling time of 2.15 (95% CI 2.03–2.29) days. Delta had the longest replacement period despite an early period of increased exponential growth relative to Omicron BA.2. After 172 days of growth, Delta Plus reached approximately 14.2% (95% CI 13.1–15.2) of all sequenced variants. This variant quickly converged to stability from exponential growth with a peak doubling time of 3.55 (95% CI 2.64–5.41) days, which is behaviour that would be expected from an additive co-existing variant.

## Discussion

Understanding the characteristics of new variants is critical to assess a growth advantage and the risk of severe disease and death. Variants of SARS-CoV-2 that have replaced the dominant variant have had a shorter incubation period, whilst co-existing variants have had very similar and non-distinct incubation period distributions relative to the dominant variant. Shorter incubation periods reflect a reduced time to peak infectiousness and therefore, a generation time advantage, which may drive replacement. The decrease in the time from hospitalisation to death and infection to hospitalisation shows that Delta caused more severe disease earlier in the course of an infection. These characteristics are important to assess risk of replacement, and thus of an epidemic wave, as well as the risk of morbidity and mortality, which may occur more quickly after infection.

The shorter incubation period provides, for a novel variant, the robust fitness difference that promotes competitive exclusion. Previous studies have found that the probability of transmission [35] and the viral load peaks [36–39] at or around symptom onset and therefore shorter incubation periods provide an earlier time to peak infectiousness. Competitive exclusion theory suggests that variants occupying the same niche cannot stably coexist and would lead to the ultimate elimination of another. Strain co-existence for SARS-CoV-2 variants can be facilitated by trade-offs between immunological evasion, cross-reactivity or a situation where there is a limited competitive transmission advantage between variants that may lead to oscillations around an equilibrium. Delta Plus showed a non-significant difference in its incubation period relative to Delta that would lead to a comparable generation time unless a substantial increase in pre-symptomatic transmission was observed. Although, the time from exposure to the pre-symptomatic infectious period appears to be related to the time to symptom onset, which has been found to be around the time when peak viral load has been measured [36–39]. Therefore, a longer incubation period would, as a result, likely lead to a longer time to the pre-symptomatic infectious period. Moreover, a longer infectious period would likely not be sufficient to provide a competitive advantage sufficient to replace a variant that had a shorter time to the peak infectious period. The suppression of Delta Plus can be seen through the lens of priority effects [40] where the advantage was given to Delta due to earlier introduction and Delta Plus did not have a transmission fitness advantage that was substantial enough to destabilise the dominant variant.

For each variant analysed in this paper, the length of the incubation period was found to be significantly related to the severity of an infection with that variant, with shorter incubation periods leading to increased risk of severe disease. However, changes in the incubation periods across variants were not predictive of severity, with variants found to have a shorter incubation period not necessarily having increased severity. Prior to the introduction of Omicron, replacing variants in England had been more clinically severe [8, 41], associated with a higher viral load and shorter incubation periods. The reduction in severity for Omicron BA.1 and Omicron BA.2 [22] may be related to viral replication being greater in the upper airways (bronchi and nasal cavity), which could also increase transmission with a shorter incubation period and an earlier pre-infectious period as a result of transmission peaking at or around the time of symptom onset [35]. Omicron, it is believed, has a replicative advantage in the nasal epithelial cells; less specialised in its cellular tropism and entering cells through the endosomal pathway. The reduction in the efficient utilisation of TMPRSS2 by the spike protein of Omicron has resulted in a reduction of syncytia formation [42–44], which may have led to diminished severity. Our results illustrate this reduced severity may also be associated with increased time to hospitalisation and death. Although, caution should be exercised to not overinterpret these laboratory-based studies that may not be applicable to a population level response, for instance, Shuai *et al*. [45] found Alpha to be the most severe variant, which was not found in this study or other population level work. This shift towards greater upper respiratory tract replication may lead to shedding characteristics that are more likely to promote earlier transmission. Therefore, we postulate that for a variant to have a competitive advantage with Omicron subvariants, it may need a similar replication advantage in the upper respiratory system or have substantially higher replication rates in the lower respiratory tract to produce earlier viral shedding that would allow for competitive transmission rates. However, replacement rates and transmission advantages of a variant are highly multifaceted and will be particularly impacted by the timing of vaccination campaigns and infection attack rates in the population.

A shorter incubation period has been illustrated across virus families [46, 47] to be related to increased severity of disease. Another study of individuals infected with the related SARS-CoV-1 virus [48] found that a shorter incubation period was associated with an increased risk of death. Our results on the relationship between the length of the incubation period and severity from SARS-CoV-2 infection are supported by Lai *et al*. [49] where a shorter incubation period was found to be associated with increased severity, defined by lung imaging (computed tomography). Further research by Cai *et al*. [50] on SARS-CoV-2 infections found an association between an incubation period of less than 7 days and severe disease. However, this study had a small sample and an improbable median of 7.48 days for the incubation period relative to results found in further studies [51–53], which may be due to data quality for the exposure and symptom onset dates and/or a lack of adjustment for the coarse nature of the collected data in analysis.

The shortest period of replacement was observed of Delta by Omicron BA.1. However, this was in an environment of limited non-pharmaceutical interventions (NPIs), and a reduction in the rate of replacement in mid-December likely reflected behavioural and policy change [3]. Delta, conversely, replaced Alpha in an environment of strict NPIs, that inhibited the rate of replacing growth, although significant spatial dispersion was observed due to a considerable number of early seeding events [2]. Relative to the earlier replacement of Delta, Omicron BA.2 replaced Omicron BA.1 at a slower rate, which is likely related to a more limited growth advantage and a high infection attack rate of Omicron BA.1 that would likely be cross reactive [54] .

Previous research [11, 12] has illustrated increased clinical severity from infections of Delta relative to Alpha and Omicron [22]. This study finds that this was associated with earlier hospitalisation and death from Delta infections. The reduction in the time from hospitalisation to death from Delta infections may also be related to the hospital admission criteria not adapting to the symptomatic presentation of novel variants with Delta patients being admitted later into the course of the infection. There is evidence that infections with Delta have a higher viral load [55] particularly in breakthrough infections [56], which will impact the incubation period as well as severity. There is presently no other evidence, outside of this study, to support whether vaccination would affect the incubation period. We found a reduced incubation period for all variants in unvaccinated individuals. Therefore, a shorter incubation period for vaccinated individuals may be indicative of reduced vaccine effectiveness.

Understanding changes to the incubation period has wider relevance to public health policy. Worldwide, quarantine periods for travellers have been enforced to limit the spread of SARS-CoV-2. Periods of quarantine have ranged from 3 days in Canada [57] to 21 days in Hong Kong [58]. Moreover, stricter policies have been enforced internationally, for example China that has pursued a zero COVID-19 policy [59]. The results illustrate for the detection of a positive case after possible exposure, a quarantine period of less than 9 days may be sufficient for the identification of 95% of cases with Omicron BA.2, however 10 days would be more suitable for Delta, Delta Plus (AY.4.2) and Omicron BA.1, with Alpha closer to 12 days. Further understanding these changes will aid in the calibration of effective public health policy.

The sample used in this study was limited to laboratory-confirmed cases that had undergone whole-genome sequencing or genotyping and the clinical case sample was determined by individuals we were able to link via a pseudo identifier. The data were not able to distinguish between types of prodromic symptoms that may offer further insight into the phenotypes of novel variants. Furthermore, the incubation period sample was restricted to the cases that were contacted by test and trace and had a symptom onset date. Previous work has illustrated that the time from hospitalisation to death is influenced by the pressure on the healthcare system due to high prevalence [27] and the epidemic phase phenomena [60, 61]. This analysis relies on contact tracing identifying the correct exposure dates. At times of low prevalence, this is likely, since it is highly probable the identified exposer is the source of infection. However, as prevalence increases, the probability of being infected by an unidentified third-party also increases. To alleviate this risk, we truncated the incubation period data at 15 days. At this level, the probability density of the incubation period distribution was sufficiently high relative to community prevalence such that the identified contact was most likely the source of infection. We found vaccination status impacted the incubation period length when analysing each variant. Therefore, the analysis of each age group may be impacted by temporally varying vaccination rates. However, we found the reduction in the length of the incubation period for replacing variants to be consistent across the age groups and by vaccination status using very large sample sizes.

## Conclusion

Novel mutations in SARS-CoV-2 associated with new variants impact the pathogenesis of the disease and replacement dynamics. The reduction in the length of the incubation period across all replacing variants suggests a mechanism for gaining competitive advantage and will be indicative of their decreased generation time. Variant replacement dynamics, however, are highly multifaceted and impacted by the unique immunological profiles of each population. We found shorter incubation periods to be associated with increased severity from an infection with a variant. However, changes in the lengths of the incubation period across variants were not found to be predictive of severity. The reduction in the time from infection to hospitalisation and hospitalisation to death illustrates the more rapid disease progression for Delta, consistent with the increased severity of this variant.

## Data Availability

To access the data used in this study, an application can be made to the UK Health Security Agency. Data requests can be made to the Office for Data Release (https://www.gov.uk/government/publications/accessing-public-health-england-data/about-the-phe-odr-and-accessing-data) and by contacting odr@phe.gov.uk. All requests to access data are reviewed by the ODR and are subject to strict confidentiality provisions in line with the requirements of:
the common law duty of confidentiality data protection legislation (including the General Data Protection Regulation) 8 Caldicott principles the Information Commissioner's statutory data sharing code of practice the national data opt-out programme.

## Supplementary material

For supplementary material accompanying this paper visit http://doi.org/10.1017/S0950268822001935. click here to view supplementary material

## Code availability

The Stan model for the doubly interval censored modelling and the instantaneous growth rate model can be found in the Supplementary material.
