# Supplementary material for: Replacement dynamics and the pathogenesis of the Alpha, Delta and Omicron variants of SARS-CoV-2
Source: Epidemiol Infect. 2022 Dec 20;151:e32. doi: 10.1017/S0950268822001935 (PMC9990386; doi:10.1017/S0950268822001935)

Supplementary A

Incubation Period

Pairwise comparisons using t tests with pooled SD

	Alpha	BA.1	BA.2	Delta
BA.1	< 2e-16	-	-	-
BA.2	< 2e-16	7.4e-09	-	-
Delta	2.4e-12	0.01317	9.3e-12	-
Delta plus	4.7e-09	0.00017	7.6e-13	1

P value adjustment method: Bonferroni

Infection to Hospitalisation

Pairwise comparisons using t tests with pooled SD

	Alpha	BA.1
BA.1	0.026	-
Delta	<2e-16	<2e-16

P value adjustment method: Bonferroni

Hospitalisation to Death

Pairwise comparisons using t tests with pooled SD

	Alpha	BA.1
BA.1	1.2e-06	-
Delta	4.1e-10	< 2e-16

P value adjustment method: Bonferroni

Supplementary B

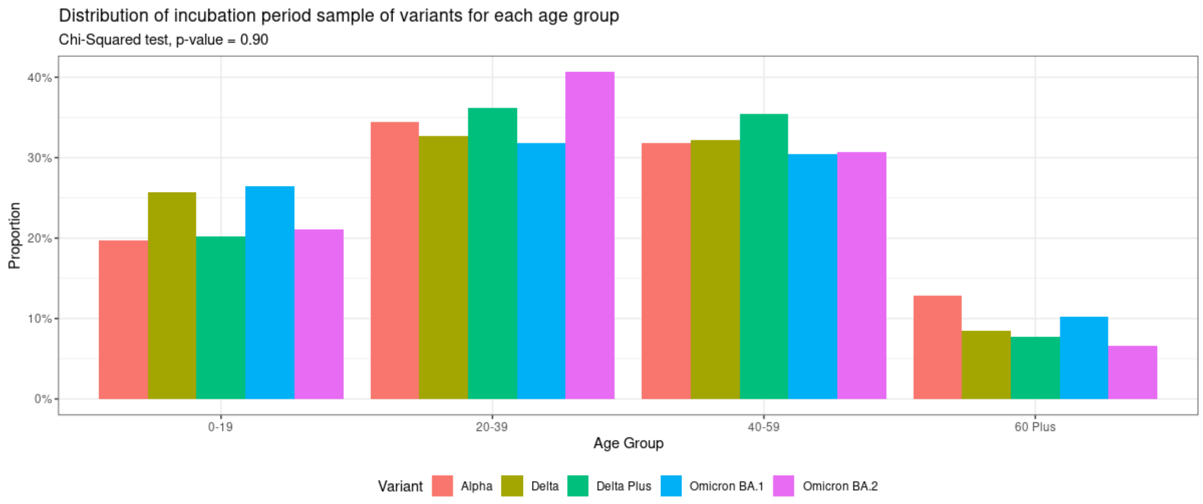

Supplementary C

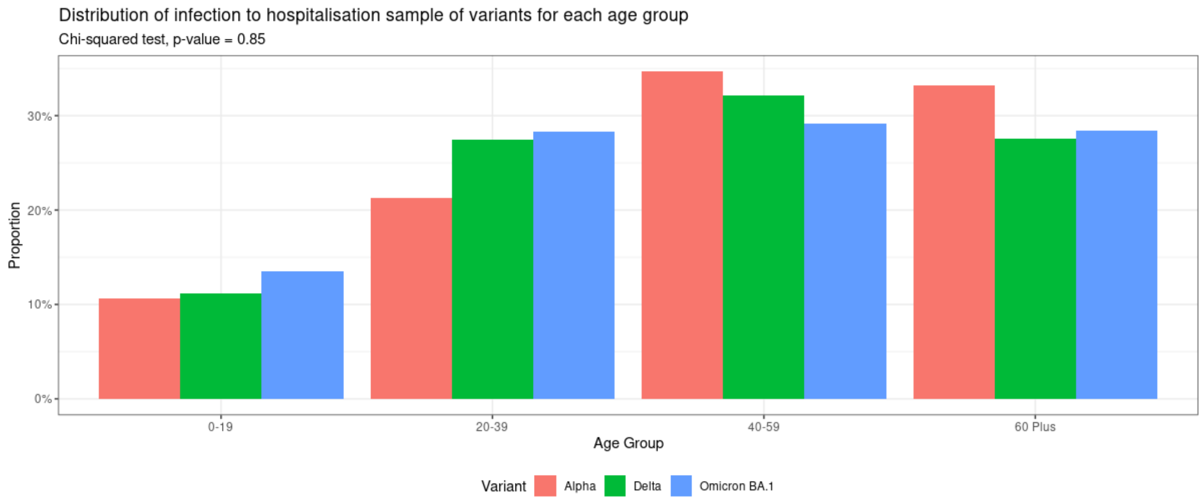

Supplementary D

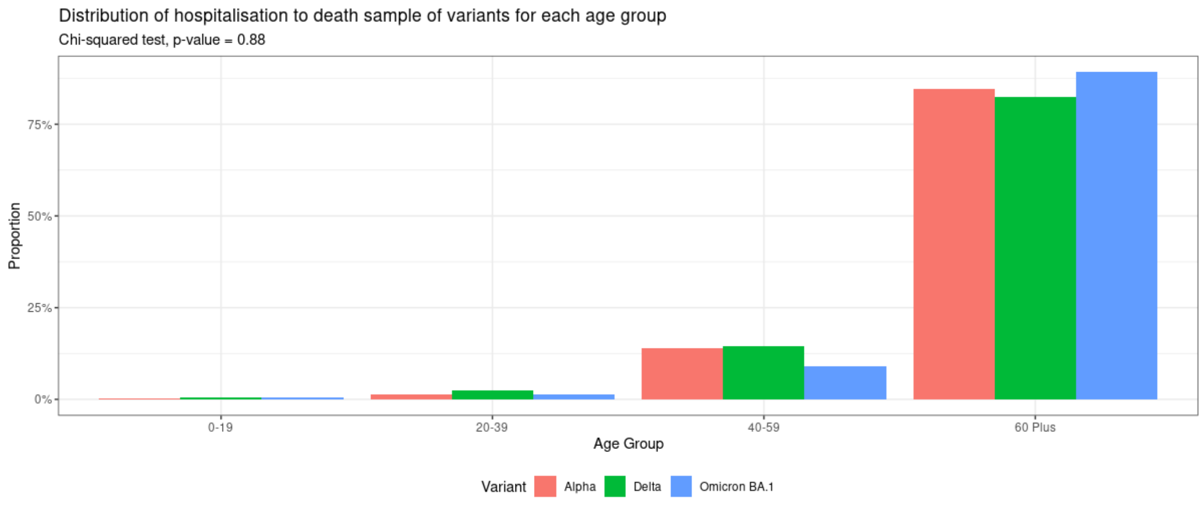

Supplement: Supplementary file 1 [file S0950268822001935sup001.pdf]
